# It Is Time to Resolve the Dilemma and Move Away From Using Drains in Primary Breast Augmentation

**DOI:** 10.1093/asjof/ojad048

**Published:** 2023-06-09

**Authors:** Paolo Montemurro, Tarush Gupta

## Abstract

**Background:**

Breast augmentation is one of the most commonly performed aesthetic surgical procedures, yet there has been no consensus on the use of drains. While some surgeons believe in using them due to fear of complications or because they were taught in a conventional manner, the authors present their experience of performing breast surgery without the use of drains.

**Objectives:**

To study whether performing breast augmentation without the use of drains is safe.

**Methods:**

Anthropometric details and complications of all the consecutive primary breast augmentation patients performed by a single surgeon from 2009 to 2022 were collected and analyzed. In none of these patients, drains were used.

**Results:**

A total of 429 (21%) patients were lost to follow-up and only those 1617 patients with a minimum follow-up of 6 months were included in this study. The mean age of the study group was 29.8 years with a mean BMI of 24.68. Mean follow-up was 16.24 months. Hematoma occurred in 15 patients (0.92%), seroma in 12 (0.74%), explantation due to infection in 3 patients (0.18%), and capsular contracture in 44 patients (2.72%). All these complications were in the lower range of complications of breast augmentation reported in the literature.

**Conclusions:**

Unwarranted use of drains in breast augmentation should be avoided as it does not seemingly prevent the complications of breast augmentation surgery. Instead, it may increase the chances of infection, pain, and discomfort, and prolong the antibiotic coverage, and hence put an additional overall financial burden on the patient.

**Level of Evidence: 4:**

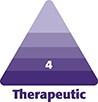

Breast augmentation is one of the most commonly requested aesthetic surgical procedures worldwide. With >364,000 operations performed in 2021, a survey conducted by the Aesthetic Plastic Surgery Society of the United States of America found it to be ranked second among all the aesthetic surgical procedures.^1^ Despite this, or perhaps because of this, there has been a concoction of views among breast surgeons on whether or not drains should be used in primary breast augmentation, with no clear consensus. One school of thought, which follows conventional teaching, is that wound drainage used after breast augmentation reduces potential complications, such as the accumulation of blood and fluid in the pocket.^2-4^ This, in turn, is assumed to decrease both short-term complications, such as hematoma and seroma, and long-term complications, especially capsular contracture, the most common complication of breast augmentation.^5^ On the other hand, many surgeons believe that drains should not be used as they involve a high cost, are time-consuming, increase the risk of infection, create physical and psychological discomfort for the patient, and do not show any benefit in the postoperative period.^6,7^ Torresetti et al performed a meta-analysis in 2020, where they could not conclude whether drains should be used in breast augmentation.^8^ Given this inconclusive view on this topic, the authors would like to present their experience with breast augmentation.

## METHODS

In our study, data were collected from all consecutive patients over the past 13 years (ie, from 2009 to 2022) operated by a single surgeon, the first author, for breast augmentation with silicone implants with an inframammary fold (IMF) access. The majority of the cases were dual plane augmentations, and the author did not use drains in any of his procedures.

### Surgical Technique and Postoperative Management

All patients are given 1 dose of antibiotic (a first-generation cephalosporin) 15 min prior to surgery, with no additional antibiotic administered postoperatively. The nipple-areola complex is covered with a transparent occlusive dressing and lidocaine-adrenaline infiltration is given at the IMF incision and in the deep planes (intramuscular or subglandular). Meticulous sharp dissection is carried out with precise proactive hemostasis. The breast pocket is then irrigated with 50% diluted betadine solution, hand gloves are changed, and betadine-irrigated breast implants are inserted using the insertion funnel. As a side note, ever since the author started using a funnel, the incision length and the capsular contracture rates have reduced compared to the procedures before its use.^9^ The closure is done in 4 layers using the first author's technique.^10^ Immediately postoperatively, compression garments are applied, and patients are discharged after 4 to 5 h.

Patients’ anthropometric details, comorbidities, and incidence of hematomas, seromas, infections, and capsular contractures in the follow-up were observed and analyzed. All procedures performed in studies involving human participants were in accordance with the ethical standards of the institutional and/or national research committee and with the 1964 Helsinki Declaration and its later amendments or comparable ethical standards. Informed consent was obtained from the patients.

## Results

A total of 2046 consecutive patients (4092 breasts) were operated on for primary breast augmentation with silicone implants over a period of 166 months, from January 1, 2009 to October 30, 2022, by the first author. A total of 429 (21%) patients were lost to follow-up, possibly (but not surely) because they never had any complication and hence did not report to us. However, in order to eliminate bias, only 1617 patients with a minimum follow-up of 6 months were included in this study. Mean follow-up was 16.24 months, ranging from 6 to 143 months. Most breast augmentations were performed in the dual plane (96.23%) and the rest in the subglandular plane (3.77%). The breast implant size varied from 140 to 615 cc, with a mean of 305.37 cc. The majority of the implants used were textured (91.1%), and the rest were smooth (8.9%). The anthropometric details and the complications in our case series are shown in Table. The mean age of the patients was 29.8 years (ranging from 18 to 62 years), and the mean BMI was 24.68. A total of 15 (0.92%) patients had postoperative hematomas. Among these, 12 patients presented within 24 h of surgery, with 3 presenting on the seventh day. All patients were taken back to the operating room; hematomas were evacuated, bleeder was controlled, and a thorough saline wash was given. None of the patients required the implant change at the time of surgery. On the long-term follow-up, none of these patients presented with capsular contracture.

**Table.** Anthropometric Details and Complications

Parameter	Value
Total number of patients	1617
Mean age	29.8 years
Mean BMI	24.68
Mean follow-up	16.24 months
Hematoma	15 (0.92%)
Seroma	12 (0.74%)
Capsular contracture	44 (2.72%)
Infection	3 (0.18%)
Implant rotation	37 (2.28%)
High riding implant	3 (0.18%)
Implant rupture	4 (0.25%)
Double bubble deformity	6 (0.37%)
Bottoming out	28 (1.73%)

The mean age of our case series was 29.8 years, with a mean BMI of 24.68. The incidence of complications like hematoma, seroma, and infections was on the lower spectrum of what was reported in the literature, in which hematoma rates were 0% to 1%, seroma rates were 0% to 2.8%, and infection rates ranged from 0% to 8.3%. Other complications like implant rotation, rupture, high riding implant, and double bubble deformity were also in the lower range of complications mentioned in the literature.^8,9,11,12^

Twelve (0.74%) patients had seroma in our series. Apart from 1 case diagnosed on the eighth day, the rest of the cases presented late, ranging from 4 to 102 months postoperatively. All these seroma cases were drained under ultrasound guidance. Among these, only 2 patients developed capsular contracture, which needed implant change. No cases of anaplastic large-cell lymphoma were found.

In our series, 44 (2.72%) patients developed capsular contracture. None of these had a history of hematoma, and only 2 cases had delayed seroma presentation. A total of 3 patients (0.18%) in our series had an infection of the site, for which the implant needed to be removed.

## DISCUSSION

Several studies have been done to investigate breast augmentation complications, which have been described in various ways. Clinically, a hematoma formation is described as a painful, tense, tight, and ecchymotic breast, which requires emergency room visit, readmission, and need for surgical procedure.^11-13^ In another study, infection was defined as redness, edema, and discharge of pus from the wound.^13,14^

A number of surgeons are convinced that using drains will help them prevent hematomas and seromas in the periprosthetic space.^2-4^ Some of them believe that the old paradigm “it is better to have them and not need them than to need them and not have them” and “when in doubt, drain” may not be tenable.^15,16^ The truth is use of drains does not prevent early surgical revisions. Hematoma collection is mainly due to bleeding from a small vessel, often a little artery, which in most cases gets detected within 24 to 48 hours after surgery. A drain is not enough to stop the bleeding and evacuate blood, and the patient needs a new surgical procedure to achieve proper hemostasis in the very majority of cases. This exploration also allows the surgeon to give a thorough wash to remove the residual hematoma collection and minimize the postexploration inflammation. Ironically, using drains can be counterproductive as the drain holes often get blocked, giving a false sense of security. Long gone are the days in which the dissection of the pocket was developed bluntly (finger dissection). This does not only increase pain in the postoperative phase but also causes fluid accumulation because of micro-trauma, with a higher risk of seroma and hematoma. Nowadays, the dissection should be done under direct view with proactive hemostasis. This enormously minimizes the pain and the amount of fluid in the pocket after the surgery.^17,18^ For this reason, sharp and meticulous dissection is an absolute prerequisite for not using the drains.

The other indication cited for using drains is for the early detection of seroma. However, the majority of surgeons prefers to take out the drains after 48 h.^8^ As seen in our case series, the chances of seroma are minimal (0.74%) in the immediate postoperative stage (seroma was observed in only 1 case). Hence, routine use of a drain will most likely not help in preventing seroma formation in primary breast augmentation. On the contrary, the presence of drains for extended periods of time may induce inflammatory reactions, causing more serous fluid production.^16^ In the authors’ opinion, drains may find their use in breast cancer cases where lymph node dissection is done, and breast implants are used for breast reconstruction.

**Figure 1.**
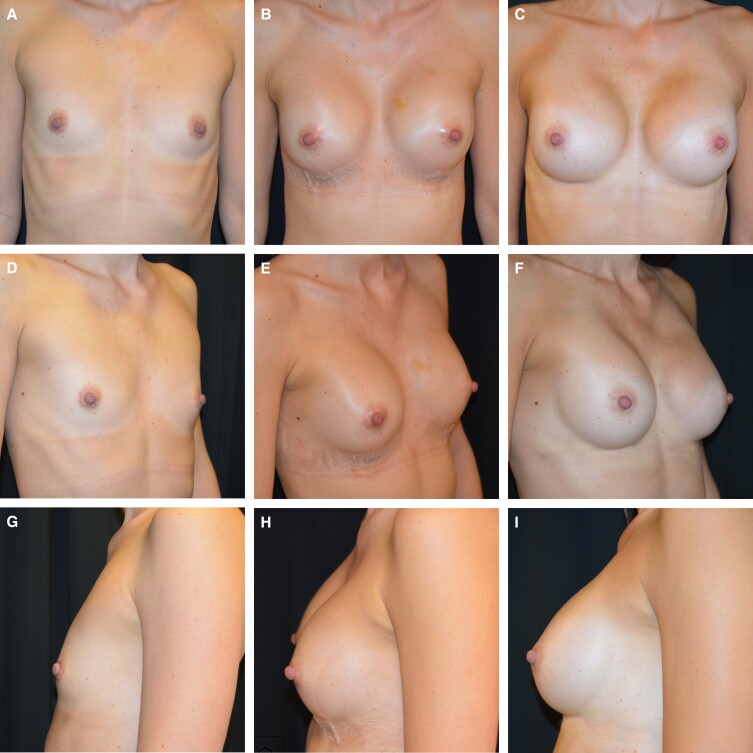
Figures represent the sequential follow-up of a 35-year-old female patient who underwent breast augmentation. Frontal, left oblique, and right laterals views are shown (A, D, G) preoperatively, (B, E, H) 1-week postoperatively, and (C, F, I) 1-year postoperatively, respectively. No drains were used in this patient. Nice breast augmentation results can be appreciated, and in the 1-week postoperative pictures, normal swelling, and the surgical tapes in situ can be seen. Patients are allowed to take a shower with these surgical tapes on the suture line from the very next day of the operation, which makes the postoperative course very comfortable. This would not be possible with the drains in situ. Aesthetically pleasing results are perfectly maintained in 1-year postoperative pictures as well.

**Figure 2.**
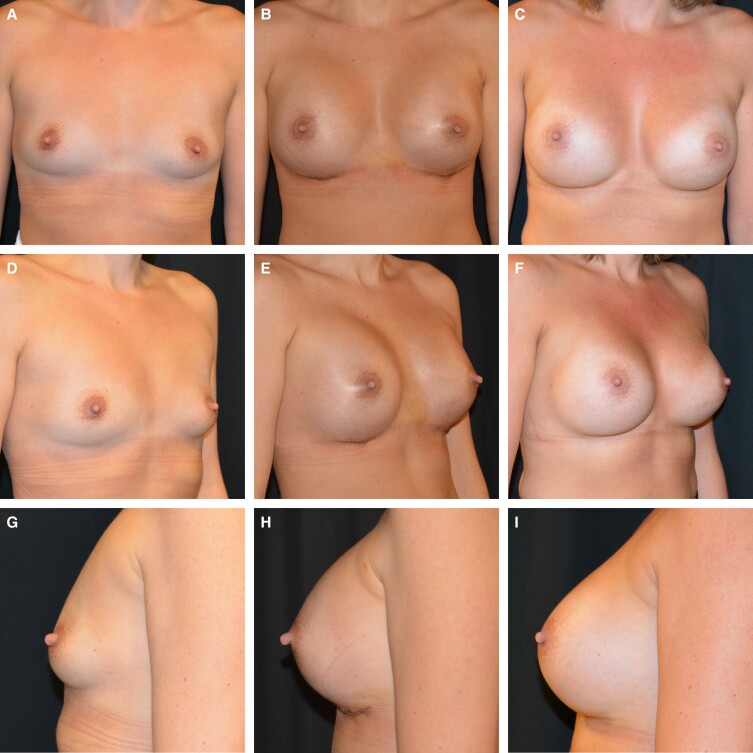
These figures represent a 32-year-old female patient with a sequential follow-up of breast augmentation at 1 week and 1 year, without the use of drains. Frontal, left oblique, and right laterals views are shown (A, D, G) preoperatively, (B, E, H) 1-week postoperatively, and (C, F, I) 1-year postoperatively, respectively. Long-term results are well maintained with good volume, projection, and symmetry.

Surgeons who are working in private setups tend to be more conservative for fear of complications. Given the increasing number of lawsuits against plastic surgeons worldwide, surgeons often feel it is safer to use drains so as to defend themselves if a patient ever sues them. Unfortunately, this practice will not only decrease the risk of complications, but also it might even increase their incidence. A thorough preoperative counseling, managing patients’ expectations, good documentation, and taking proper care of complications, if they occur, will certainly prevent more lawsuits against surgeons than the use of drains.^19,20^Figures 1 and 2 show the preoperative views of 2 patients before breast augmentation, without the use of drains, sequentially followed up with 1-week postoperative and 1-year postoperative pictures.

The use of drains has also other disadvantages. It is a potential site of infection. Studies have found that the chances of infection increase with the use of drains owing to bacterial colonization of the drainage tubes.^21,22^ Most surgeons feel more comfortable providing antibiotic coverage until the drain is in situ.^23^ This prolongs the use of antibiotics, prolongs the hospital stay, increases the overall financial burden on the patients, and ends up using more hospital resources. Injudicious use of antibiotics will also create more antibiotic resistance, which is a serious problem world is facing in the present times.^24^ Lastly, the physical discomfort and pain of carrying the drains and dressing around can cause psychological distress in the patient. In the world of aesthetic surgery, where our motive is to provide a comfortable and early pain-free experience, unwarranted drains go entirely against this principle.

The limitation of our study is the lack of a control group, as none of the patients was operated with drains to compare with. Despite this, our complications are in line, if not lower, than those reported in the literature.^8^ Therefore, while we cannot state that no drains are superior to using drains, we can for sure claim that not using them will not cause more complications. The strength of our study is the big case series with over 13 years of meticulous record keeping of consecutive patients, operated by a single surgeon, which eliminates the bias from multiple techniques adopted by different surgeons.

## CONCLUSIONS

The field of surgery has witnessed tremendous advances in the past decades, with the techniques getting refined and the use of evidence-based practices bringing significant improvement in patient management. Plastic surgery is no different, and surgeons should not keep on operating the same way as a few decades ago. The use of drains in primary breast augmentation is not wrong per se, but making it a norm is. We need to evolve and refine ourselves for the sake of patients. Drains give no benefit; if anything, they can actually harm, and many surgeons keep using them just because they were taught so and do not dare to change their routines. We must look beyond drains in primary breast augmentations to provide patients with a better quality of life.
